# Exploring the Relationship Between Lifestyle Choices and Menstrual Health

**DOI:** 10.7759/cureus.114009

**Published:** 2026-08-05

**Authors:** Annika N Lundberg, Iman A Majid, Alana M Cruickshank, Pierce Imperialbobis, Valeria Nieri, Jane Hufnagel, Madison R McCraney, Chandler Pendleton, Sarah Alvarez

**Affiliations:** 1 Clinical Sciences, Florida State University College of Medicine, Tallahassee, USA; 2 Biomedical Sciences, Florida State University College of Medicine, Tallahassee, USA

**Keywords:** fast food frequency, high risk behaviors, lifestyle, menstrual irregularity, menstruation, reproductive health

## Abstract

Introduction: Many international studies have worked to understand the relationship between lifestyle factors, like nutrition and risky behaviors, and menstrual health in young women. This study focuses on understanding the relationship between lifestyle choices and period irregularity in adolescents and young adult women in the United States specifically.

Methods: This prospective study utilized an online survey that was distributed to women ages 10-25 years old at the North Florida Pediatrics clinic and Florida State University in Tallahassee, Florida. The primary endpoint focused on analyzing the relationship between fast food frequency and menstrual health characteristics, including menstrual period regularity, menstrual period duration, and menstrual cycle length. Secondary endpoints focused on analyzing the relationship between menarche and risky behaviors, including drug and alcohol use. Exclusion criteria included a personal history of polycystic ovary syndrome (PCOS), endometriosis, or other menstrual-related conditions and current birth control use.

Results: A total of 90 responses were collected from the survey and used for data analysis. After implementing exclusion criteria, responses from 29 adolescent and young women were used for analysis of the relationship between fast food frequency and menstrual health. No statistically significant association was found between fast food frequency and period regularity (p=0.7), length of menstrual cycle (p=0.66), and length of menstrual period (p=0.16). Due to a limited sample size (n=15), analysis between menarche and sexual activity was performed, but demonstrated no statistically significant relationship (p>0.9).

Discussion: Our data did not show a statistically significant relationship between the menstrual variables and lifestyle factors surveyed. Further research should be completed to investigate the relationship between fast food consumption and menstrual abnormalities. Additional lifestyle factor endpoints, such as tobacco and alcohol use or exercise frequency, should be investigated to better understand the impact of habits on menstruation.

## Introduction

Menarche, or the age at which a female will start their menstrual cycle, is an important step in the development of a woman. The median age of menarche is between 12 and 13 years [1]. Since the 20th century, the age of menarche has been declining [2]. Different factors may affect the timing including socioeconomic status, nutrition, and genetics [2]. Furthermore, menstrual irregularities are common in adolescent females as the hypothalamic-pituitary-ovarian axis matures, making it difficult to ascertain true gynecologic conditions in young females. Menstrual irregularities can cause a decline in the quality of life of young women and have negative outcomes on their sexual and mental health as well as academic performance [3]. In addition, the stigma surrounding menstrual health can act as an obstacle to accessing healthcare and lead to undiagnosed clinical conditions [3]. The American College of Obstetricians and Gynecologists recommend using the menstrual cycle as a vital sign in young adolescents and females, encouraging providers and parents to have more open and engaging conversations about menstrual health [1].

Adolescence is a period of rapid change in which young people establish behavioral patterns that may have lasting consequences on their overall health [4]. One of the most pivotal changes of adolescence include the transition through puberty, characterized by physical growth as well as emotional and hormonal changes [5]. In females, the primary changes in development include thelarche, pubarche, and menarche [5]. While ovulatory dysfunction may be physiological in the first few years after menarche due to the immature hypothalamic-pituitary axis, there are a variety of factors that may lead to period irregularity. These include nutrition (obesity, rapid weight change, underweight), stress, hyperadrenergic anovulation, and other hormonal imbalances [1].

The rapid growth and maturation of adolescence is a time period that requires additional nutrients and energy-rich foods [6]. Given the importance that nutrition plays in overall health, poor dietary habits are a potential factor influencing menstrual irregularity [7]. From August 2021 to 2023, the CDC reports that those aged 1-18 years of age consumed 61.9% of their total calories in ultra-processed foods. These foods tend to be hyperpalatable, energy-dense foods containing large amounts of sodium, sweetener, and fats [8]. A study done in Sweden found that childhood nutritional status was related to height changes and early menstruation [9]. With an increasing culture of over-consumption in America, early intervention in adolescent nutrition can have lasting impacts on their overall health outcomes.

The association between diet, obesity, and menstrual health has been well documented. In a cross-sectional study conducted in Turkey, researchers surveyed Turkish women 18-65 years of age analyzing socio-demographic characteristics, nutritional habits, and menstrual status. The study found that women with menstrual disorders were more likely to have lower intake of vitamins and protein when compared to healthy women. In addition, they are more likely to consume high-sugar food and beverages [10]. A recent prospective study conducted in the U.S. investigated the relationship between diet and age at menarche. Researchers found that a healthier diet was associated with a later age of menarche, and similarly an inflammation-associated diet was associated with earlier menarche [11]. Although the relationship between diet and obesity has been well established in the literature, the association between increased fast food intake and menstrual health has not been well documented in the context of the American diet. In a cross-sectional study conducted at Bahria International Hospital in Lahore, researchers collected information on frequency of junk food consumption, baseline data, and menstrual pattern in girls aged 13-19. They found that consumption of refined grains, sugary foods, beverages, and fast foods were associated with menstrual abnormalities (p<0.05) [12]. Studies on the United States fast food diet and menstrual health are limited, and this study aims to contribute to the literature to better understand a possible link between American fast food consumption and abnormalities in menstrual health.

As age of menarche is a critical milestone in development, it may influence other lifestyle behaviors that many young people are introduced to throughout their adolescence and into adulthood such as sexual behavior (age of sexual debut, number of sexual partners, and use of protection) [13,14] and substance use patterns (alcohol, nicotine, tobacco product, and marijuana use) [15,16]. While parents often categorize these behaviors as “risky”, they are also directly associated with significant changes in health outcomes due to prolonged lifetime exposure to health risks [17]. Specifically, earlier sexual debut has been linked to increased number of lifetime sexual partners, increased rate of sexually transmitted infections, and unintended pregnancy [18]. Similarly, earlier onset of substance use extends the duration of exposure to carcinogens and other harmful substances found in nicotine/tobacco products, alcohol, and marijuana [19]. Similar to the primary outcome of this study, the associations between early menarche and risky behaviors has been well established in studies conducted outside of the United States. By examining these behaviors as a secondary outcome in relation to age of menarche, this may be able to provide a framework for developing targeted screening and prevention tools in adolescents using their menstrual history as an additional identifier for the implementation of these strategies.

The primary outcome of this study is to better understand the relationship between lifestyle factors and menstrual abnormalities, specifically fast food consumption, in adolescents and young adult women in the United States. We will test our hypothesis that based on other studies of its kind, increased fast food consumption is associated with menstrual irregularity. Although well described internationally, the relationship between fast food intake and menstrual health is not well established in the United States. Secondary outcomes include the relationship between early menarche and risky behaviors, such as substance use and sexual behaviors. Our hypothesis is such that early menarche and risky behaviors are positively associated. Through this study, we aim to raise the awareness of how certain lifestyle choices can impact the health of adolescent females in the U.S. and in turn highlight the importance of early intervention. Preliminary findings from this study were presented as two poster presentations at the American Medical Women’s Association’s Region 4 Conference on January 17, 2026.

## Materials and methods

Data collection

An anonymous survey was created on Qualtrics (Qualtrics, LLC, Provo, Utah, and Seattle, Washington, USA) to investigate the relationship between lifestyle factors and menstrual health. The study and survey questions were approved via the Institutional Review Board of Florida State University, Tallahassee, USA (approval number: STUDY00005985, date: April 30, 2025). This survey was distributed on Florida State University’s campus and in the North Florida Pediatrics clinic located in Tallahassee, Florida, via flyers and social media. The data collection period was from April 2025 to December 2025. Individuals were recruited via physical flyers and through social media platforms including GroupMe (Microsoft, New York City, NY, USA) and Instagram (Meta Platforms Menlo Park, California, USA).

There were two versions of the survey. These separate versions ensured age-appropriate questions, as data regarding risky behaviors were included for the adult participants. Version 1 was filled out by patients aged 10-17 years old. This version assessed the patients’ menstrual cycle and fast food consumption. Version 2 was distributed to patients 18 to 25 years old and included additional questions to assess risky behaviors. The risky behavior questions obtained information regarding the participant’s sexual activity and drug and alcohol use. Both versions collected information on menstrual cycles, including length, age of menarche, intermittent bleeding, and heaviness of flow. A question relating to fast food frequency was adapted from the National Health and Nutrition Examination Survey (NHANES) Diet, Behavior, and Nutrition Questionnaire [20]. Appendix 1 and Appendix 2 demonstrate both versions of the surveys that were distributed to participants. The survey was completely voluntary with no monetary reward provided. No personal health information was collected.

Participants

Females ages 10-25 years old were included in the survey. Patients with underlying medical conditions were excluded from the study. The exclusion criteria included those that are pregnant, those with polycystic ovarian syndrome, and individuals taking oral contraceptives. This criteria was implemented as these factors can contribute to menstrual irregularity and may serve as confounding variables. Incomplete survey responses were also excluded in sections if they were relevant for specific data analysis sections. Parental consent was required for participants under 18 years of age, and we requested the minor individual to complete the survey to ensure self-reported data reflected the patient’s experience.

Analytical approach

Descriptive statistics are presented for the entire sample including participants that have a diagnosis of PCOS, use birth control, and/or are pregnant; only participants that did not answer Q2: “If you are interested in completing this survey, please indicate which age range you/your child belongs to so that you can be redirected to the appropriate consent page” were excluded from the descriptive analysis (n = 1). The mean and standard deviation are presented for all continuous variables; the frequency and proportion are presented for all categorical variables.

Wilcoxon rank-sum tests were used to test associations between binary variables and frequency of fast food consumed in the past seven days; Spearman rank correlation was used to test monotonic associations between numeric variables and frequency of fast food consumed in the past seven days. Exact p-values could not be calculated due to ties in the data. No adjustments were made for multiple comparisons. Bivariate analysis exploring a possible association between menarche and being sexually active was conducted using a Wilcoxon rank-sum test. Exact p-values could not be calculated due to ties in the data.

## Results

Sample size and exclusion

A total of 91 participants completed the Qualtrics survey. One participant was excluded from the statistical analysis, as they did not complete the consent page appropriately. Regarding the Demographics section, 26 participants did not complete the survey section in its entirety and were excluded from the demographic analysis section of our research (n=64). Regarding the Lifestyle section of the survey for participants <18 years old, 18 participants did not complete the question relating to fast food frequency (n=19), and 14 participants (greater than 18 years old) did not complete questions relating to risky behaviors, and their responses were excluded from data analysis (n=39). Regarding the Menstrual Health section, only 49 participants completed the section in its entirety.

Due to variations in responses to our survey, the sample size used for the analysis of our endpoints was significantly smaller than the initial sample size. Exclusion criteria for understanding the relationship between fast food frequency and menstrual irregularity included incomplete responses to either sections, personal history of PCOS, endometriosis, or other menstrual-related conditions, and current birth control use. With the application of our exclusion criteria, the relationship between fast food frequency and menstrual irregularity had a sample size of n=29. Our analysis of the relationship between menarche and sexual activity had a sample size of n=15.

Demographics

The age of participants was categorized into three ranges: 10-13 years old, 14-17 years old, and 18+ years old. Of the 90 survey responses, 21% (n=19) of participants fell into the 10-13 year old category, 20% (n=18) fell into the 14-17 year old category, and 59% (n=53) of participants were 18 years or older. Table 1 displays the information relating to racial identity and family annual household income.

**Table 1:** Racial Identity (n=70) and Annual Household Income (n=64) of Participants.

Race	Number of Participants (n=70)	Percentage
Asian	7	10
African American	7	10
Hispanic	8	11.4
Middle Eastern	2	2.9
White	46	65.7
Household Income	Number of Participants (n=64)	Percentage
Less than $20,000	2	3.1
$20,000-$74,999	15	23
$75,000-$99,999	14	22
More than $100,000	27	42
Not Sure	6	9.4

Fast food frequency

Among the 19 participants younger than 18 years old, the mean number of meals eaten from a fast food or pizza place in the past seven days was 3 (SD=4). Of the 35 participants that were 18 years or older, the mean number of meals eaten from a fast food or pizza place in the past seven days was 3 (SD=3).

Menstrual health

Of the 54 participants that were eligible to complete the menstrual health section, five were excluded due to incomplete responses (n=49). The mean age of menarche was 12 years old (SD=1). Regarding menstrual health abnormalities, 18.4% (n=9) of participants experienced a menstrual cycle more or less than once a month. The mean length of participants' menstrual cycle was 21 days (SD=21) and the mean length of their menstrual period was seven days (SD=6). Intermenstrual bleeding was reported in 6.1% (n=3) of participants. Regarding diagnosis of PCOS, endometriosis, or any other menstrual-related conditions, 10% (n=5) of participants reported a personal history and 12% (n=6) of participants reported a family history. Specific results relating to participants' menstrual health is shown in Table 2.

**Table 2:** Menstrual Characteristics of Participants (n=49). PCOS: polycystic ovary syndrome.

Menstrual Characteristics (n=49)	Mean (SD)	Number of Participants	Percentage
Age of menarche (years)	12 (1)		
Length of menstrual cycle (days)	21 (12)		
Length of last menstrual period (days)	7 (6)		
Frequency of Menstrual Period			
More than twice a month		2	4.1
Twice a month		2	4.1
Once a month		40	82
Every other month		2	4.1
Less than every month		3	6.1
Frequency of Changing Menstrual Products			
<2 hours		2	4.1
Every 3-5 hours		32	65
Every 6-8 hours		15	31
Intermenstrual Bleeding		3	6.1
Personal History of PCOS, Endometriosis, etc.		5	10
Family History of PCOS, Endometriosis, etc.		6	12
Currently Pregnant		0	0
Use Hormonal Birth Control		16	33

Risky behaviors

Of the 53 participants that were 18 years or older, 14 participants did not complete the lifestyle section in its entirety and were excluded (n=39). Nicotine use was reported by 5.2% (n=2) of participants. Marijuana use was reported by 7.7% (n=3) of participants. Alcohol use was reported by 66% (n=26) of participants. About 44% (n=17) of participants reported engaging in sexual activity. Table 3 shows detailed results relating to the frequency of risky behaviors. Table 4 demonstrates the average age participants started engaging in risky behaviors, in addition to other characteristics.

**Table 3:** Frequency of risky behaviors among participants 18 years or older (n=39).

Risky Behaviors	Number of Participants (n=39)	Percentage
Frequency of Nicotine Use		
Not at all	37	95
Less than monthly	1	2.6
Monthly	0	0
Weekly	1	2.6
Daily	0	0
Prefer not to say	0	0
Frequency of Marijuana Use		
Not at all	35	90
Less than monthly	3	7.7
Monthly	0	0
Weekly	0	0
Daily	0	0
Prefer not to say	1	2.6
Frequency of Alcohol Use		
Not at all	6	15
Less than monthly	11	28
Monthly	0	0
Weekly	15	38
Daily	0	0
Prefer not to say	7	18
Frequency of Sexual Activity		
Not at all	12	31
Less than monthly	8	21
Monthly	0	0
Weekly	9	23
Daily	0	0
Prefer not to say	10	26

**Table 4:** Risky Behavior Characteristics Among Participants 18 Years or Older.

	Mean (SD)	Minimum, Maximum	Number of Participants	Percentage
Nicotine (n=2)				
Age of onset (years)	19 (1)	18-19		
Length of time participant used nicotine products (years)	5 (1)	4-5		
Currently use nicotine products			0	0
Marijuana (n=3)				
Age of onset (years)	19 (2)	18-21		
Alcohol (n=26)				
Age of onset (years)	18 (2)	15-22		
Sexual Activity (n=17)				
Age of onset (years)	18 (2)	15-22		
Use condoms				
Yes			9	53
Sometimes			4	24
No			3	18
Prefer not to say			1	5.9

Fast food frequency and menstrual irregularity

After excluding incomplete responses, personal history of PCOS, endometriosis, and other menstrual-related disorders, and current hormonal birth control usage, responses from 29 participants were used to examine associations between fast food frequency and menstrual irregularity. There was no statistically significant relationship between fast food frequency and menstrual period frequency (p=0.7), length of menstrual cycle (p=0.66), and length of menstrual period (p=0.16). Table 5 demonstrates specific results relating to the relationship between fast food frequency and period frequency. Table 6 details the statistical analysis for the correlation between length of menstrual cycle and period and fast food frequency.

**Table 5:** No Statistically Significant Correlation Between Fast Food Frequency and Period Frequency (p=0.7).

	More or Less Than Once a Month (n=6), Mean (SD); (min,max)	Once a Month (n=25) Mean (SD); (min,max)	p-Value
Number of meals eaten from fast food or pizza place in the past 7 days	5 (6); (1,18)	3(2); (0,8)	0.7
Missing (n=5)			

**Table 6:** No Statistically Significant Relationship Between Fast Food Frequency and Menstrual Period Length (p=0.16) and Menstrual Cycle Length (p=0.66).

	Rho	p-Value
Length of Menstrual Cycle	-0.08	0.66
Length of Menstrual Period	0.27	0.16

Menarche and risky behaviors

Due to so few participants answering questions relating to risky behaviors, only a statistical analysis exploring the relationship between menarche and sexual activity was conducted (n=15). There was no statistical significance between menarche and sexual activity (p>0.9). Details of this analysis can be visualized in Table 7. 

**Table 7:** No Statistically Significant Relationship Between Menarche and Sexual Activity.

	Not Sexually Active (n=8) Mean (SD); (min,max)	Sexually Active (n=7) Mean (SD); (min,max)	p-Value
Menarche	12 (1); (11,14)	12 (2); (9,14)	>0.9

## Discussion

This study was conducted due to a lack of research that recognizes menstrual abnormalities and their association with lifestyle factors. This study sought to investigate if such associations existed in order to advance research that helps educate female adolescents and young adults about how lifestyle decisions impact their menstrual health. This study is one of the first of its kind in the United States and was inspired by international studies that looked at similar variables. This study’s purpose was to stimulate future research on how various lifestyle factors may impact young women’s health in order to better educate patients and parents.

In our study, the mean number of meals eaten from fast food or pizza places in the past seven days was 3. About 18.4% (n=9) of individuals had their period more or less than once a month, which characterizes an irregular period. About 6.1% (n=3) of individuals experienced intermenstrual bleeding. About 10% (n=5) of women noted that they had been diagnosed with PCOS, endometriosis, or any other menstrual-related condition, and 12% (n=6) noted a family history of such conditions.

When the association between the number of meals eaten from fast food or pizza places in the past seven days and period frequency (once a month vs. more or less than once a month) was analyzed, no statistically significant difference was found (p=0.7). Additionally, there was no statistically significant relationship between fast food frequency and menstrual cycle length (p=0.66) and menstrual period length (p=0.16). This is in contrast to a survey conducted in India that found a positive association between increased junk food consumption and menstrual disorders [7]. Furthermore, a cross-sectional study in Maharajganj used the Healthy Eating Assessment Tool Score and determined that 68.6% of participants (mean age 24.56±2.65 years) ate fast food and 91.6% of those students experienced menstrual abnormalities, with increased fast food intake frequency positively associated with increased menstrual problems [21]. Additionally, a cross-sectional study conducted in Lahore found that fast food consumption was significantly associated with menstrual abnormalities (p<0.05) [12]. These studies suggest that our results are distinct and should be replicated with more insight into dietary habits and a larger sample size.

Additionally, the relationship between menarche and sexual activity was not statistically significant (p>0.9). This is in contrast to previous literature, which suggests a relationship between the variables. An in-person interview-style study conducted in Tanzania found that a younger age of menarche was associated with earlier onset of sexual activity (p=0.009) [14]. Additionally, a Korean survey found that early menarche was associated with higher risk of early-onset sexual activity and risky behavior [22]. Thus, it may be important to correlate our findings with additional surveys of larger populations and geographic areas.

Our study’s drawbacks include the geographic limitations to Tallahassee, Florida, meaning that the results cannot be generalized to all young females. The study sample size was also small, so results should be interpreted with caution. A type 2 error should be considered when interpreting relationships due to the decrease in sample size, which further decreased the statistical power of the data. Additionally, our study is a cross-sectional, observational survey, and thus, our results cannot show causation but should encourage future randomized controlled studies to better understand the impact of lifestyle choices on menstruation. Furthermore, our study used short, simple questions for adolescents to maintain focus and adherence to the survey. Our study did not use a validated questionnaire or assessment tool for our survey, although future research may help develop a tool to make inter-study analysis comparisons more feasible. Future studies should study specific variable metrics such as diet specifications and fast food franchises to better understand individuals’ lifestyles and draw more specific conclusions. Limitations of this study include a small sample size, limited geographic area, and anonymous survey structure that may introduce erroneous data points. Future research should focus on repeating surveys targeting a larger sample size and geographic area, extending survey questions to study specific types of processed foods, creating a controlled experiment to test data points, and evaluating the extent to which lifestyle choices impact menstrual health.

## Conclusions

The goal of this paper was to describe an association between menstrual abnormalities and lifestyle factors. This paper did not find a statistically significant association between these variables among females aged 10-25 years old at North Florida Pediatrics or Florida State University in Tallahassee, Florida. This finding highlights the need to encourage future research that investigates how lifestyle choices may impact menstruation.

## Appendices

Appendix 1 

**Table 8:** Version 1: Survey for Females Aged 10-17 Years Old.

Question Number	Question	Responses						
	Demographics							
1	Were you assigned female at birth?	Yes	No					
2	How old are you?	Numerical answer (years)						
3	What is your race or ethnicity?	Asian	African American	Hispanic	Middle Eastern	Native American	White	Prefer not to say
4	What is your household income?	Under $20,000	$20,000-$74,999	$75,000-$99,000	More than $100,000			
5	What is your current weight (in lbs)?	Numerical answer						
6	What is your current height (in ft)?	Numerical answer						
	Lifestyle							
7	Please indicate the number of meals you have eaten from fast-food or pizza place in the past 7 days?	Scale from 0-21						
	Menstrual Health							
8	Have you ever had a menstrual period?	Yes	No					
	Questions 9-14 will appear if participant answers "Yes" to question 8							
9	How old were you when you first started your period?	Numerical Answer	Have not started yet					
10	How often do you have your period?	More than twice a month	Twice a month	Once a month	Every other month	Every two months or more	Other	
11	How long (in days) is your menstrual cycle? (Menstrual cycle is defined as time from the first day of your menstrual period until the first day of your next menstrual period)	Numerical Answer						
12	How long (in days) was your last menstrual period?	Numerical answer						
13	During your menstrual period, how often do you have to change your tampon or pad?	<2 hours	Every 3-5 hours	Every 6-8 hours				
14	Do you bleed in between each period?	Yes	No					
15	Have you been diagnosed with polycystic ovary syndrome (PCOS) or any other menstrual-related conditions?	Yes	No					
16	Is there a family history of polycystic ovary syndrome (PCOS), endometriosis or other menstrual-related conditions?	Yes	No					
17	Do you use hormonal birth control?	Yes	No					
18	Are you currently pregnant?	Yes	No					

Appendix 2 

**Table 9:** Version 2: Survey for Females Aged 18 Years and Older.

Question Number	Question	Responses						
	Demographics							
1	Were you assigned female at birth?	Yes	No					
2	How old are you?	Numerical answer (years)						
3	What is your race or ethnicity?	Asian	African American	Hispanic	Middle Eastern	Native American	White	Prefer not to say
4	What is your household income?	Under $20,000	$20,000-$74,999	$75,000-$99,000	More than $100,000			
5	What is your current weight (in lbs)?	Numerical answer						
6	What is your current height (in ft)?	Numerical answer						
	Lifestyle							
7	Please indicate the number of meals you have eaten from fast-food or pizza place in the past 7 days?	Scale from 0-21						
8	How often do you engage in these activities?							
	i. Nicotine	Never	Once	Less than monthly	Monthly	Weekly	Daily	Prefer not to answer
	ii. Marijuana	Never	Once	Less than monthly	Monthly	Weekly	Daily	Prefer not to answer
	iii. Alcohol	Never	Once	Less than monthly	Monthly	Weekly	Daily	Prefer not to answer
	iv. Sexual activity	Never	Once	Less than monthly	Monthly	Weekly	Daily	Prefer not to answer
	Questions 9-11 will appear if participant does not answer "not at all" for nicotine products							
9	How old were you when you first used nicotine products (cigarette, vape, etc.)?	Numerical scale	Prefer not to answer					
10	How long have you used any nicotine-based products (cigarette, vape, etc.)?	Numerical scale	Prefer not to answer					
11	Are you currently using any nicotine products (cigarette, vape, etc.)?	Yes	No	Prefer not to answer				
	Question 12 will appear if participant does not answer "not at all" for marijuana							
12	How old were you when you first used marijuana?	Numerical scale	Prefer not to answer					
	Question 13 will appear if participant does not answer "not at all" for alcohol:							
13	How old were you when you started drinking alcohol?	Numerical scale	Prefer not to answer					
	Questions 14-15 will appear if participant does not answer "not at all" for sexual activity:							
14	At what age did you become sexually active?	Numerical scale	Prefer not to answer					
15	Do you use condoms?	Yes	Sometimes	No	Prefer not to answer			
	Menstrual Health							
16	Have you ever had a menstrual period?	Yes	No					
	Questions 17-26 will appear if participant answers "Yes" to question 8							
17	How old were you when you first started your period?	Numerical Answer	Have not started yet					
18	How often do you have your period?	More than twice a month	Twice a month	Once a month	Every other month	Every two months or more	Other	
19	How long (in days) is your menstrual cycle? (Menstrual cycle is defined as time from the first day of your menstrual period until the first day of your next menstrual period)	Numerical Answer						
20	How long (in days) was your last menstrual period?	Numerical answer						
21	During your menstrual period, how often do you have to change your tampon or pad?	<2 hours	Every 3-5 hours	Every 6-8 hours				
22	Do you bleed in between each period?	Yes	No					
23	Have you been diagnosed with Polycystic Ovary Syndrome (PCOS) or any other menstrual-related conditions?	Yes	No					
24	Is there a family history of Polycystic Ovary Syndrome (PCOS), endometriosis or other menstrual-related conditions?	Yes	No					
25	Do you use hormonal birth control?	Yes	No					
26	Are you currently pregnant?	Yes	No					
